# Supplementary material for: Hypocretinergic interactions with the serotonergic system regulate REM sleep and cataplexy
Source: Nat Commun. 2020 Nov 27;11:6034. doi: 10.1038/s41467-020-19862-y (PMC7699625; doi:10.1038/s41467-020-19862-y)
Supplement: Supplementary file 1 — Supplementary Information [file 41467_2020_19862_MOESM1_ESM.pdf]

Hypocretinergic interactions with the serotonergic system regulate REM sleep and cataplexy

Ali Seifinejad, Sha Li, Marie-Laure Possovre, Anne Vassalli[1]*, and Mehdi Tafti[1]*

Department of Biomedical Sciences, Faculty of Biology and Medicine, University of Lausanne, 1005 Lausanne, Switzerland

[1]These authors contributed equally

***Corresponding Authors**: Prof. Mehdi Tafti, Department of Biomedical Sciences, Faculty of Biology and Medicine, University of Lausanne, Rue du Bugnon 7, 1005 Lausanne, Switzerland, Phone:+41215923971, Fax: +41216925505, email: mehdi.tafti@unil.ch.

Dr. Anne Vassalli, Department of Biomedical Sciences, Faculty of Biology and Medicine, University of Lausanne, Rue du Bugnon 7, 1005 Lausanne, Switzerland, email: anne.vassalli@unil.ch.

Keywords: Narcolepsy, lateral hypothalamus, dorsal raphe, serotonin transporter

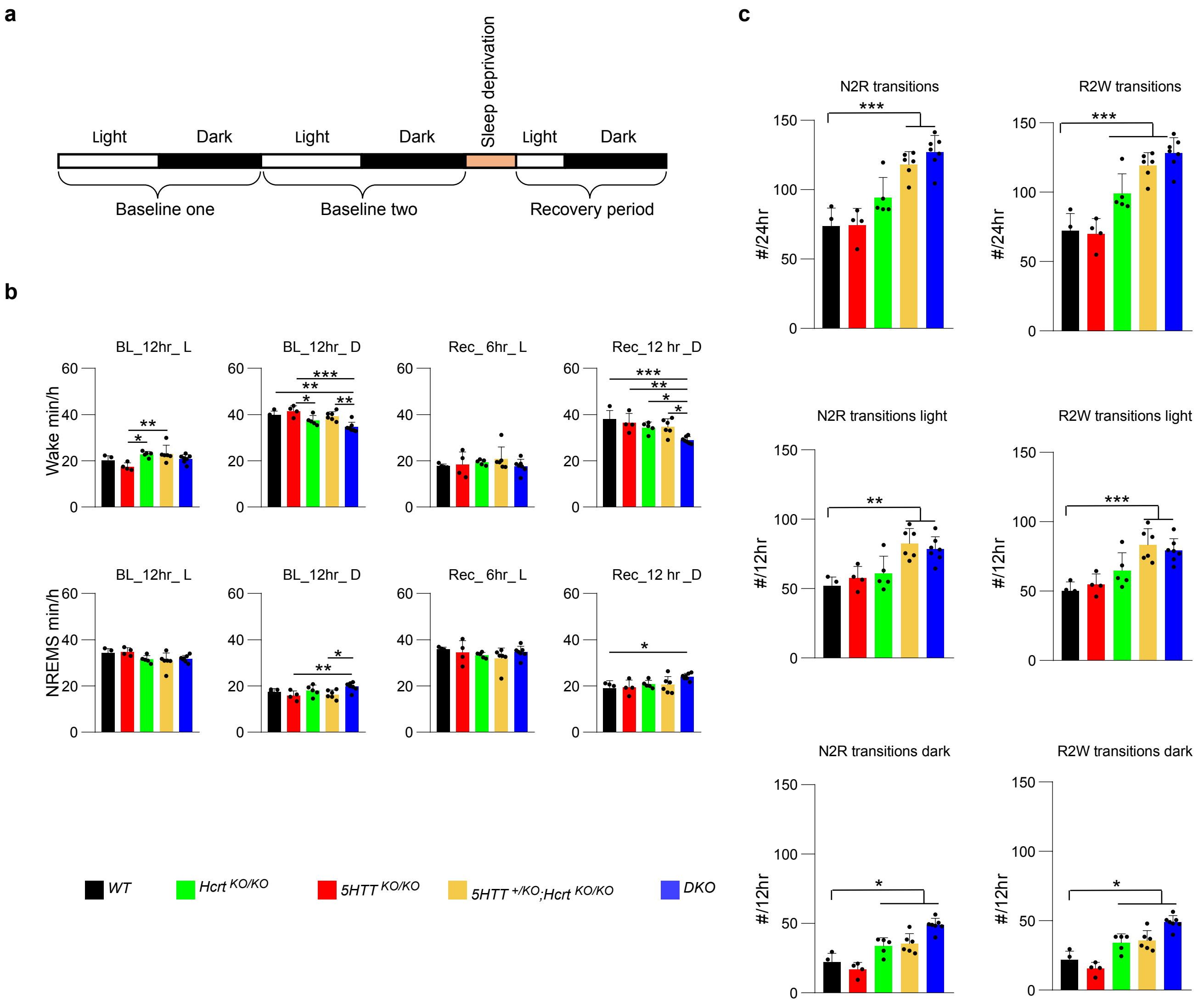

Supplementary Fig. 1. a Schematic figure representing the experimental procedure. **b** Total amount of wakefulness and NREMS across genotypes during light and dark periods (one-way ANOVA, genotype F (4,21) = 4.49 (wake, BL light, P < 0.01), 9.94 (wake, BL dark, P < 0.001), 0.73 (wake, Rec light, P > 0.5) and 7.35 (wake, Rec dark, P < 0.001), 3.1 (NREM, BL light, P < 0.05), 3.6 (NREM, BL dark, P < 0.05), 1.05 (NREM, Rec light, P > 0.4) and 3.23 (NREM, Rec dark, P < 0.05) followed by Tukey test, mean ± SD. L: light, D: dark, BL: baseline, Rec: recovery **c** Number of NREMS to REMS (N2R) and REMS to Wake (R2W) state transitions across genotypes and during light and dark periods. L: light, D: dark, BL: baseline, Rec: recovery. Values are mean ± SD (one-way ANOVA, genotype F (4, 21) = 21.59 (N2R-24hr), 26.3 (R2W-24hr), 9.5 (N2R-light), 10.8 (R2W-light), 23.6 (N2R-dark), and 24.9 (R2W-dark), P < 0.0001, followed by Tukey test). For b and c * P < 0.05; ** P < 0.01; *** P < 0.001.

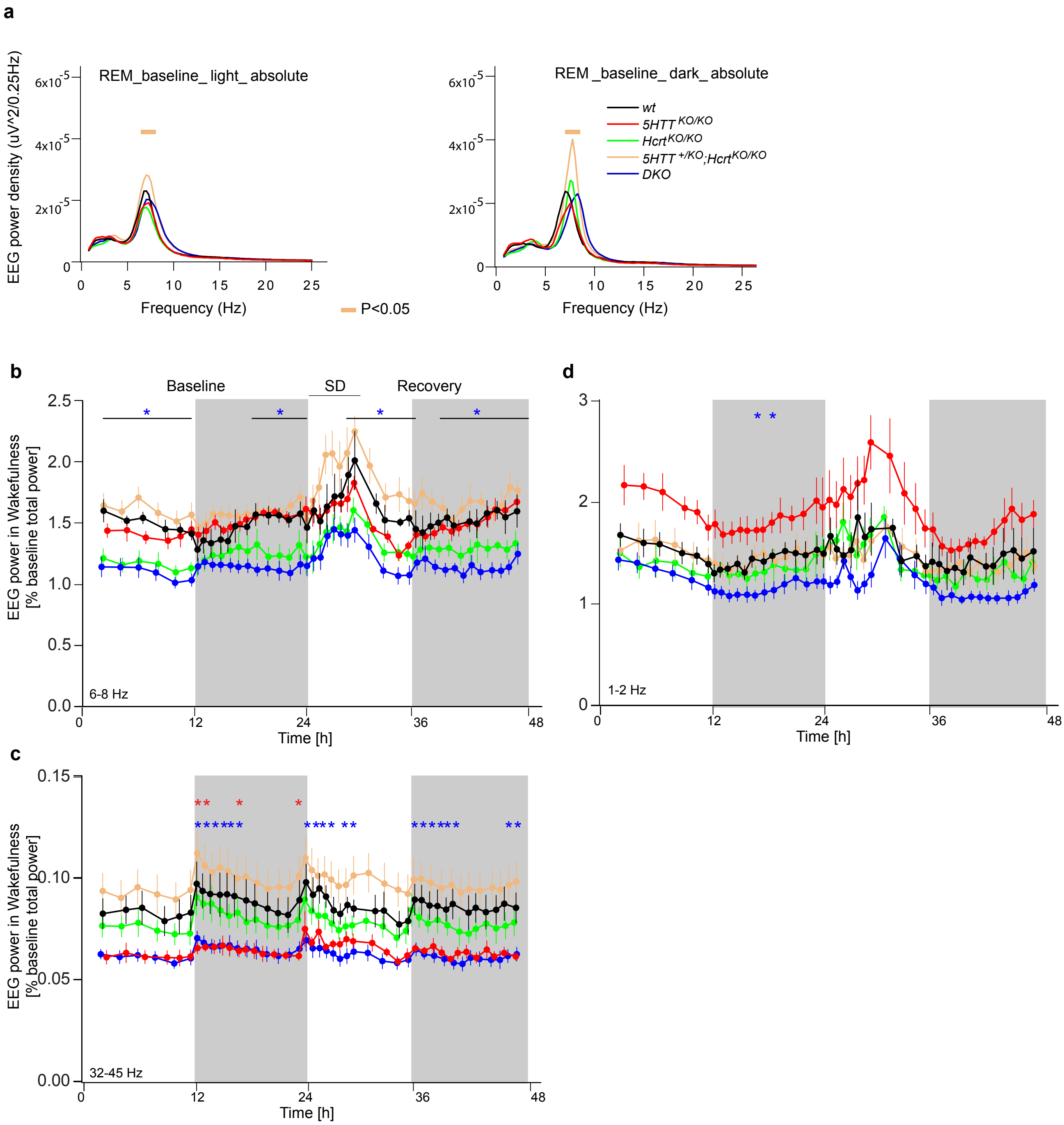

Supplementary Fig. 2. a Absolute baseline REMS EEG power spectra among genotypes during light (left) and dark (right) periods (two-way ANOVA, interaction frequency x genotype F (388, 2037) = 1.59 (light), 3.3 (dark), P < 0.0001, followed by Dunnett's test). **b** Time-course dynamics of the theta (6-8 Hz) EEG power in wakefulness (two-way ANOVA, interaction time x genotype F (164, 861) = 1.83, P < 0.0001, followed by Dunnett's test, mean ± SD). **c** Time-course dynamics of the slow-gamma (32-45 Hz) EEG power in wakefulness (two-way ANOVA, genotype F (4, 21) = 5.66, P <0.01, followed by Dunnett's test, mean ± SD). **d** Time-course dynamics of the slow-delta (1-2 Hz) EEG power in wakefulness (two-way ANOVA, genotype F (4, 21) = 5.04, P < 0.01, followed by Dunnett's test, mean ± SD), WT vs 5HTT+/KO;HcrtKO/KO *, DKO *, 5HTTKO/KO *, HcrtKO/KO * P < 0.05.

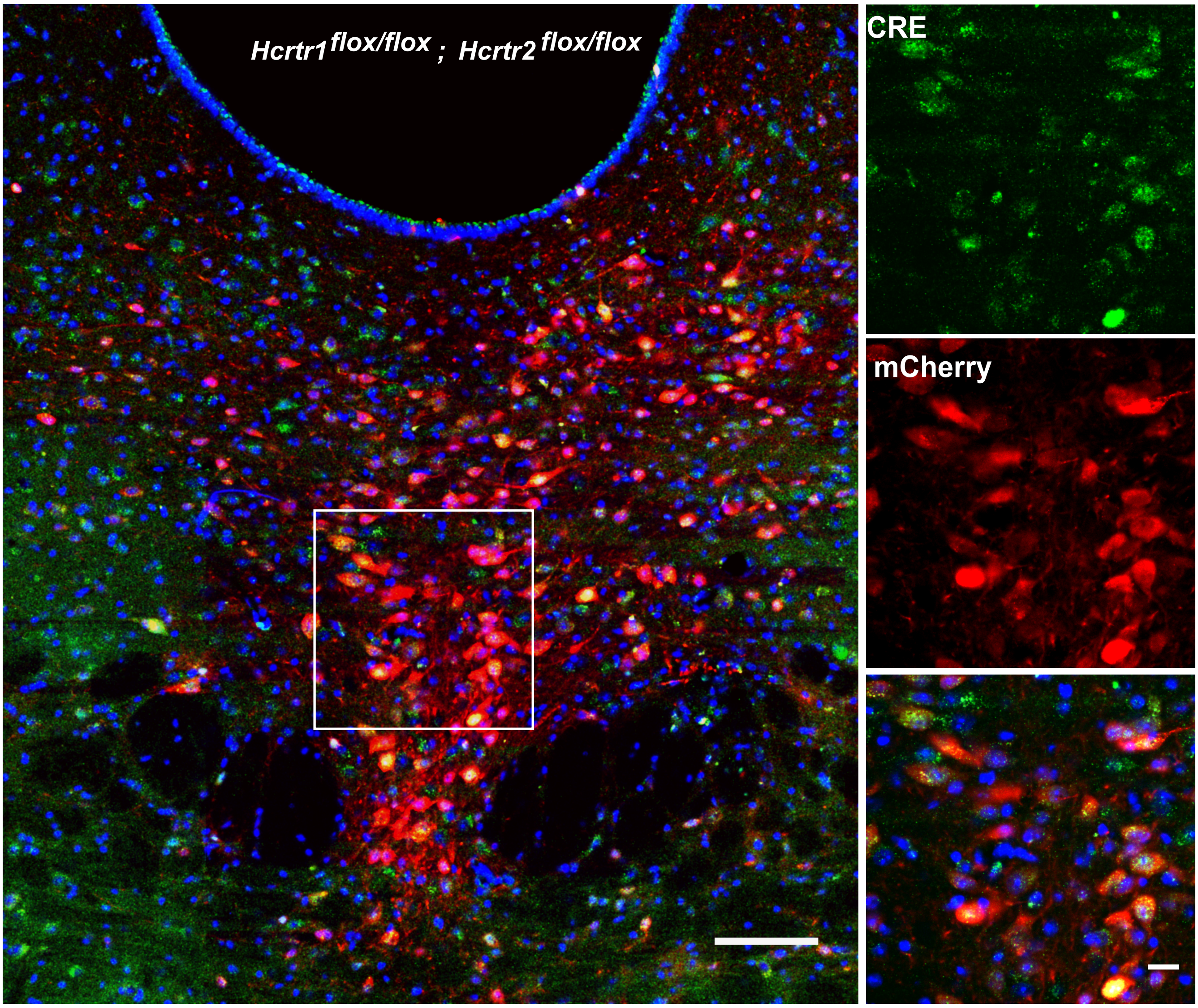

Supplementary Fig. 3. Colocalization of mCHERRY and Cre expression in the DR of HCRTr1&2 flox/flox mice. Representative confocal image of the DR of Hcrtr1&2 double-floxed homozygous mice injected with AAV-EF1a-mCherry-IRES-Cre-WPRE and stained with mCHERRY (red) and Cre (green) antibodies. mCHERRY-positive cells (red) are highly co-localized with Cre-positive cells (green). Higher magnification (40X, right) indicates substantial Cre expression in mCHERRY-positive cells. Scale bars: main 100 m, magnification 20 m.

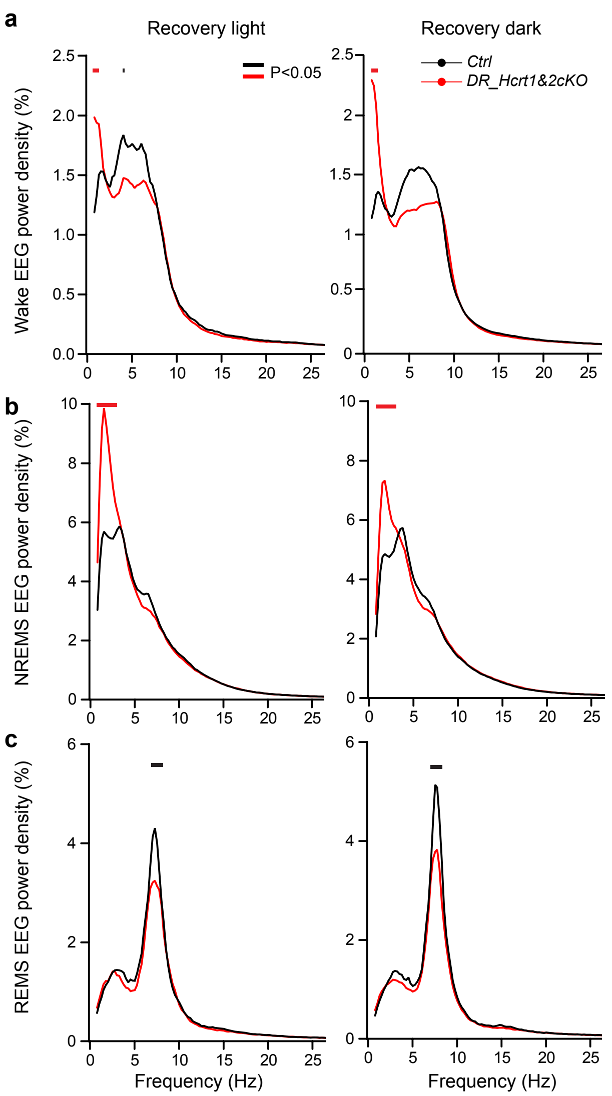

Supplementary Fig. 4. Alterations in vigilance states EEG power spectra in DR Hcrtr1&2 gene-inactivated mice. **a** Waking EEG power spectra during light (left) and dark (right) periods during recovery. The data are normalized to the total EEG power during baseline. **b** EEG power spectra for NREMS during recovery. c EEG power spectra for REMS during recovery. Values are mean ± SEM (two-way ANOVA, frequency x genotype F (196, 1568) = 3.05 (wake light), 5.63 (NREMS light), 1.85 (REMS light), 2.7 (wake dark), 2.39 (NREMS dark) and 4.75 (REMS dark), P <0.0001, followed by Dunnett's test, P < 0.05).

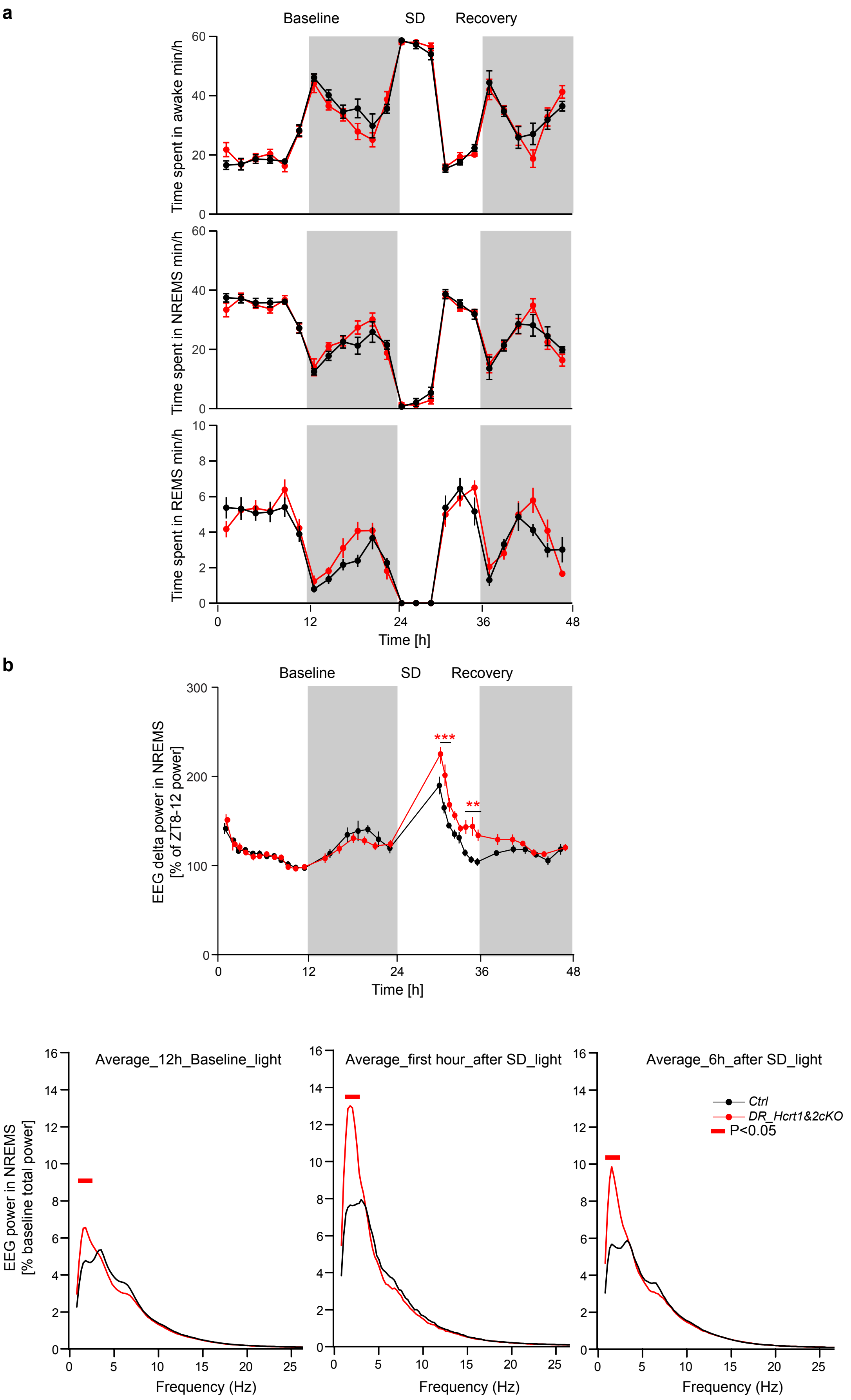

Supplementary Fig. 5. **a** Time-course distribution of vigilance states after inactivation of Hcrtr1&2 in the DR, Wakefulness (top), NREMS (middle) and REMS (bottom). Data points are shown in minutes spent in each state per 1 hour interval. Baseline represents the average of 2 days of recordings before sleep deprivation (SD), (DR_HcrtR1&2-cKO n=5, and control mice n=5, mean ± SEM). **b** Time-course of the EEG delta power (top, two-way ANOVA, interaction time x genotype $F(31, 248) = 3.69$, $P < 0.0001$, followed by Dunnett's test, ** $P < 0.01$; *** $P < 0.001$, and total EEG power (bottom) in 12 hours baseline, first hour after SD and 6 hours after SD. Values are mean ± SEM (two-way ANOVA, interaction frequency x genotype $F(196, 1568) = 2.26$ (left), 3.90 (middle), and 5.63
(right), $P < 0.0001$, followed by Dunnett's test).
