# Supplementary material for: Hypocretinergic interactions with the serotonergic system regulate REM sleep and cataplexy
Source: Nat Commun. 2020 Nov 27;11:6034. doi: 10.1038/s41467-020-19862-y (PMC7699625; doi:10.1038/s41467-020-19862-y)
Supplement: Supplementary file 3 — Reporting Summary [file 41467_2020_19862_MOESM3_ESM.pdf]

nature research

Corresponding author(s): Mehdi Tafti
NCOMMS-19-39487C

Last updated by author(s): Oct 29, 2020

Reporting Summary

Nature Research wishes to improve the reproducibility of the work that we publish. This form provides structure for consistency and transparency in reporting. For further information on Nature Research policies, see Authors & Referees and the Editorial Policy Checklist .

Statistics

For all statistical analyses, confirm that the following items are present in the figure legend, table legend, main text, or Methods section.

n/a	Confirmed	
☐	☒	The exact sample size (n) for each experimental group/condition, given as a discrete number and unit of measurement
☐	☒	A statement on whether measurements were taken from distinct samples or whether the same sample was measured repeatedly
☐	☒	The statistical test(s) used AND whether they are one- or two-sided<br>*Only common tests should be described solely by name; describe more complex techniques in the Methods section.*
☐	☒	A description of all covariates tested
☐	☒	A description of any assumptions or corrections, such as tests of normality and adjustment for multiple comparisons
☐	☒	A full description of the statistical parameters including central tendency (e.g. means) or other basic estimates (e.g. regression coefficient) AND variation (e.g. standard deviation) or associated estimates of uncertainty (e.g. confidence intervals)
☐	☒	For null hypothesis testing, the test statistic (e.g. F, t, r) with confidence intervals, effect sizes, degrees of freedom and P value noted<br>*Give P values as exact values whenever suitable.*
☒	☐	For Bayesian analysis, information on the choice of priors and Markov chain Monte Carlo settings
☒	☐	For hierarchical and complex designs, identification of the appropriate level for tests and full reporting of outcomes
☒	☐	Estimates of effect sizes (e.g. Cohen's d, Pearson's r), indicating how they were calculated

Our web collection on statistics for biologists contains articles on many of the points above.

Software and code

Policy information about availability of computer code

Data collection	EMBLATM hardware was used for signal acquisition and Somnologica- 3TM (Medcare) software for data analysis. High-resolution CCD cameras (Panasonic WV-CP500) were used for high-quality, horizontal (side-view) video recording.
Data analysis	Signal processing and data analysis was performed using custom written algorithms in MATLAB R2015a software. GraphPad prism 8 was used for statistical analysis.

For manuscripts utilizing custom algorithms or software that are central to the research but not yet described in published literature, software must be made available to editors/reviewers. We strongly encourage code deposition in a community repository (e.g. GitHub). See the Nature Research guidelines for submitting code & software for further information.

Data

Policy information about availability of data

All manuscripts must include a data availability statement. This statement should provide the following information, where applicable:

- Accession codes, unique identifiers, or web links for publicly available datasets
- A list of figures that have associated raw data
- A description of any restrictions on data availability

We (the authors) declare that the data supporting the findings of our study are available within the paper and its supplementary information files.

Field-specific reporting

Please select the one below that is the best fit for your research. If you are not sure, read the appropriate sections before making your selection.

☒ Life sciences ☐ Behavioural & social sciences ☐ Ecological, evolutionary & environmental sciences

Life sciences study design

All studies must disclose on these points even when the disclosure is negative.

Sample size	we determined the sample size based on experiences during last 25 years of animal experimentation work and our published literatures (Vassalli et al, 2013 brain, Maret et al, 2007, PNAS). We did not use statistical test to determine sample size.
Data exclusions	Animals without correct, enough and local expression of viral particles were removed from analysis. Animals with good EEG, EMG and video recording were kept for the analysis and the rest were excluded. Good EEG, EMG and video recording means that any mice with high quality EEG but disrupted EMG and vice versa and also the same for video recording were excluded. This step was done before moving to analysis part. During video analysis, animals without normal behavior (e.g. sick behavior), were excluded from the study.
Replication	Under investigation
Randomization	All genotypes were randomly distributed for recording. This means that in each seri of recording we had different combination of the genotypes. Vigilance state scoring was done blindly.
Blinding	People who performed sleep deprivation and viral injection were totally blind to the genotype of the animals. In the analysis the investigators were blind during the EEG scoring.

Reporting for specific materials, systems and methods

We require information from authors about some types of materials, experimental systems and methods used in many studies. Here, indicate whether each material, system or method listed is relevant to your study. If you are not sure if a list item applies to your research, read the appropriate section before selecting a response.

Materials & experimental systems

n/a	Involved in the study	
☐	☒	Antibodies
☒	☐	Eukaryotic cell lines
☒	☐	Palaeontology
☐	☒	Animals and other organisms
☒	☐	Human research participants
☒	☐	Clinical data

Methods

n/a	Involved in the study	
☒	☐	ChIP-seq
☒	☐	Flow cytometry
☒	☐	MRI-based neuroimaging

Antibodies

Antibodies used	Antibodies used are mouse-anti-TPH (sigma, Cat# T0678), Rat-anti-mCHERRY (life technologies, Cat# M11217) and Chicken-anti-GFP (Aves Labs, Cat# 1020), Rabbit-anti-CRE (Novagen, Cat# 69050-3).
Validation	The antibodies that we used, all are commercially-available, and were used according to manufacturer's instructions.<br>mCherry Antibody (M11217) in IF<br>U2OS cells were transduced using an adenoviral construct expressing mCherry. A) Native expression of mCherry detected post-transduction using Texas Red filters (562 nm/624 nm) B) Anti-Cherry antibody added and cells imaged using the Cy5 filter set (628 nm/692 nm) C) mCherry expression detected by adding anti-mCherry and Alexa Fluor® 647 goat anti-rat (Product # A-21247).<br><br>Anti-checken-GFP: Antibodies were analyzed by western blot analysis (1:5000 dilution) and immunohistochemistry (1:500 dilution) using transgenic mice expressing the GFP gene product. Western blots were performed using BlokHen® (Aves Labs) as the blocking reagent, and HRP-labeled goat anti-chicken antibodies (Aves Labs, Cat. #H-1004) as the detection reagent. Immunohistochemistry used tetramethyl rhodamine-labeled anti-chicken IgY.<br><br>Rabbit-anti-CRE: Evaluated by Western Blotting with Recombinant Cre-His. Western Blotting Analysis: Representative lot data. Recombinant Cre-His loaded at 20 ng/lane (Lane 1) and 10 ng/lane (Lane 2) was probed with Cat. No. 69050-3, Anti-Cre (1:10,000 dilution). Proteins were visualized using a Donkey Anti-Rabbit secondary antibody conjugated to HRP and a chemiluminescence detection system. Arrow indicates Cre (~39 kDa).<br><br>Validation of TPH antibody has been reported in 68 published papers based on the company website.

Animals and other organisms

Policy information about studies involving animals; ARRIVE guidelines recommended for reporting animal research

Laboratory animals	the following mouse lines were used in our study:<br>Hcrt knock out mice (Chemelli, 1999)<br>5HTT knouk out mice (Bengel, 1998)<br>Hcrtr1/Hcrtr2 double-floxed mice (Vassalli et al, 2015; Li et al 2018).<br><br>All analyzed mice were male and at the age between 12-14 week old.
Wild animals	No wild animals were used in this study.
Field-collected samples	No field collected samples were used in this study.
Ethics oversight	All animal procedures followed Swiss federal laws and were approved by the State of Vaud Veterinary Office.

Note that full information on the approval of the study protocol must also be provided in the manuscript.
